# Switchable Nanozyme Activity of Porphyrins Intercalated in Layered Gadolinium Hydroxide

**DOI:** 10.3390/ijms232315373

**Published:** 2022-12-06

**Authors:** Maria A. Teplonogova, Marina V. Volostnykh, Alexey D. Yapryntsev, Madina M. Sozarukova, Yulia G. Gorbunova, Ekaterina D. Sheichenko, Alexander E. Baranchikov, Vladimir K. Ivanov

**Affiliations:** 1 Kurnakov Institute of General and Inorganic Chemistry of the Russian Academy of Sciences, 119991 Moscow, Russia; 2 Frumkin Institute of Physical Chemistry and Electrochemistry of the Russian Academy of Sciences, 119071 Moscow, Russia; 3 Faculty of Chemistry, National Research University “Higher School of Economics”, 109028 Moscow, Russia

**Keywords:** layered rare-earth hydroxides, porphyrins, hybrid material, nanozyme, antioxidants, prooxidants, radical scavenging properties, chemiluminescence

## Abstract

In this study, organo-inorganic nanohybrids **LHGd-MTSPP** with enzyme-like activity were prepared by in situ intercalation of anionic 5,10,15,20-tetrakis-(4-sulfonatophenyl)porphyrin and its complexes with Zn(II) and Pd(II) (**MTSPP**, M = 2H, Zn(II) and Pd(II)) into gadolinium layered hydroxide (**LHGd**). The combination of powder XRD, CHNS analysis, FT-IR, EDX, and TG confirmed the layered structure of the reaction products. The basal interplanar distances in **LHGd-MTSPP** samples were 22.3–22.6 Å, corresponding to the size of an intercalated tetrapyrrole molecule. According to SEM data, **LHGd-MTSPP** hybrids consisted of individual lamellar nanoparticles 20–50 nm in thickness. The enzyme-like activity of individual constituents, **LHGd-Cl** and sulfoporphyrins **TSPP**, **ZnTSPP** and **PdTSPP**, and hybrid **LHGd-MTSPP** materials, was studied by chemiluminescence analysis using the ABAP/luminol system in phosphate buffer solution. All the individual porphyrins exhibited dose-dependent antioxidant properties with respect to alkylperoxyl radicals at pH 7.4. The intercalation of free base **TSPP** porphyrin into the **LHGd** preserved the radical scavenging properties of the product. Conversely, in **LHGd-MTSPP** samples containing Zn(II) and Pd(II) complexes, the antioxidant properties of the porphyrins changed to dose-dependent prooxidant activity. Thus, an efficient approach to the design and synthesis of advanced **LHGd-MTSPP** materials with switchable enzyme-like activity was developed.

## 1. Introduction

Natural enzymes are efficient catalysts, possessing high activity and selectivity, which play an important role in biological reactions [1]. However, the poor stability of enzymes in harsh environments, complicated preparation processes, and the high cost of production significantly limit their practical application [2]. Substantial research efforts have been made to develop artificial enzymes as an alternative to natural enzymes, to mimic the catalytic activity of the latter [1].

Nanozymes are artificial nanomaterials with intrinsic enzyme-like properties. As a new generation of synthetic enzymes, nanozymes have attracted considerable attention, owing to their advantages: facile synthesis, versatility, low cost, good chemical stability, and high catalytic activity [3,4,5]. The first paper devoted to nanozymes was published as early as 2007 [6]. In that study, Fe_3_O_4_ nanoparticles possessed peroxidase-like activity similar to that of natural peroxidases. Since the publication of this study, the field of nanozyme research has progressed rapidly. It was shown that nanozymes can act as antioxidants and protect living cells from oxidative damage by participating in redox reactions and the scavenging of reactive oxygen species (ROS) [7]. Enzyme-like activities are inherent in metal oxide nanoparticles [8,9], carbon nanomaterials [10,11], metal–organic frameworks [12], porphyrin-based porous organic polymers [13], and layered double hydroxides (LDHs) [1,14].

Layered metal hydroxides are a family of compounds consisting of positively charged stacked hydroxide layers with anionic species in the interlayer spacing [15]. Currently, layered hydroxides include the most widely studied LDHs, layered simple hydroxides, and layered rare-earth hydroxides (LRHs) [16,17,18,19]. Although LRHs are close analogues of LDHs [19], they contain rare-earth cations that are responsible for additional functionality. For instance, rare-earth-containing layered hydroxides exhibit bright UV-Vis and NIR luminescence (Eu^3+^, Gd^3+^, Tb^3+^, etc.) [20,21,22,23]. Layered gadolinium hydroxide (**LHGd**) nanosheets have been used as effective contrast agents for magnetic resonance imaging [24].

The anion-exchange properties of layered hydroxides enable facile modification and fine tuning of their functional characteristics, including their catalytic, photophysical, and magnetic properties. Recently, it was demonstrated that these materials are able to immobilise enzymatic and molecular antioxidants [25,26,27,28]. Among antioxidant species, several phenolic compounds were intercalated into LRHs, e.g., ascorbic acid, ferulic acid, and gallic acid. For example, gallate was found to preserve its antioxidant character upon intercalation in MgAl-LDH [29] and ZnAl-LDH [30].

Porphyrins and related macrocycles are photosensitisers with rich photochemical and photophysical properties [31,32,33,34]; they are used as catalysts for the oxidation of various organic compounds [35,36,37], as singlet oxygen sources for photodynamic therapy [38,39,40], and in different types of sensors [41], etc. Over the past few decades, the antioxidant activity of porphyrins has been extensively investigated due to the unique redox properties of a macrocyclic conjugated π-system. It was found that the nature of the metal centre and peripheral substituents is a very important factor for dictating porphyrin antioxidant activity and efficiency [42,43]. For example, cationic Mn(III) and Fe(III) N-substituted pyridylporphyrins [44,45,46] and tetrasubstituted porphyrins functionalised with antioxidant di-*tert*-butylphenol groups were shown to be efficient antioxidants and biomimetics of superoxide dismutase [42,44,47].

Zhang et al. [48] and Qiao et al. [49] prepared peroxidase mimetic composites based on iron-containing natural porphyrin–hemin, and FeNi- and CuAl-LDHs. It was shown that hemin is a good candidate for intercalation, owing to its planar structure and negative charge through the carboxylic groups. It was demonstrated that FeNi(hemin)-LDH possesses excellent peroxidase-like activity in comparison with a free hemin. This was attributed to the dimerisation of hemin outside the layered material; while in intercalated form, the hemin is mostly monomeric. CuAl(hemin)-LDH, designed by Qiao et al., also exhibited good antioxidant activity and proved to be a good scavenger of peroxynitrite ions ONOO^−^ [49].

Anionic sulfoporphyrins can easily be intercalated into layered hydroxides. For example, the preparation of hybrid materials based on LDHs and 5,10,15,20-tetrakis-(4-sulfonatophenyl)porphyrin (TSPP) and its complexes with Zn(II) and Pd(II) was reported recently, along with an investigation of their photophysical properties [50,51,52,53,54,55]. It was shown that intercalated porphyrins do not aggregate in the interior of layered hydroxides, and can act as efficient photosensitisers producing singlet oxygen O_2_(^1^∆_g_) [50,53,54].

Despite this, only one paper has been devoted to the formation of LRH-sulfoporphyrin hybrids [56]. In this paper, layered europium hydroxide and layered terbium hydroxide were intercalated with TSPP or PdTSPP. The LEuH-TSPP material demonstrated the ability to form O_2_(^1^Δ_g_) upon excitation in an oxygen atmosphere, thus demonstrating the redox regulating property of such hybrids and indicating their possible enzyme-like activity.

Taking into account the enzyme-like behaviour of the materials based on layered double hydroxides and redox activity of porphyrins, the present work was focused on the radical scavenging property of sulfoporphyrins intercalated in layered rare-earth hydroxides (namely, layered gadolinium hydroxide) being the closest chemical analogues of LDHs. For this, a series of hybrid materials based on layered gadolinium hydroxide **LHGd** and 5,10,15,20-tetrakis-(4-sulfonatophenyl)-porphyrins **MTSPP** (M = 2H, Zn(II), Pd(II)) was prepared by hydrothermal synthesis and characterized with a wide range of analytical methods. The radical scavenging property and enzyme-like activity of the layered rare-earth hydroxides were demonstrated for the first time.

## 2. Results and Discussion

### 2.1. Preparation and Characterisation of LHGd Intercalated with Porphyrin

Layered hydroxides intercalated with organic anions are generally synthesised using the following approaches: anion exchange, coprecipitation at a constant pH [19,57], and rehydration [58] methods. Contrary to the other methods for layered hydroxides synthesis, anion exchange and coprecipitation techniques consist of one or two synthetic steps only, which makes them the most commonly used. In the current work, for the preparation of a layered gadolinium hydroxide intercalated with *meso*-tetra(4-sulfonatophenyl)porphyrin (**LHGd-TSPP**), two synthetic strategies were applied (anion exchange and coprecipitation), combined with hydrothermal treatment, ensuring good crystallinity of the products. The comparison of the samples obtained by these approaches is presented below.

**Figure 1:**
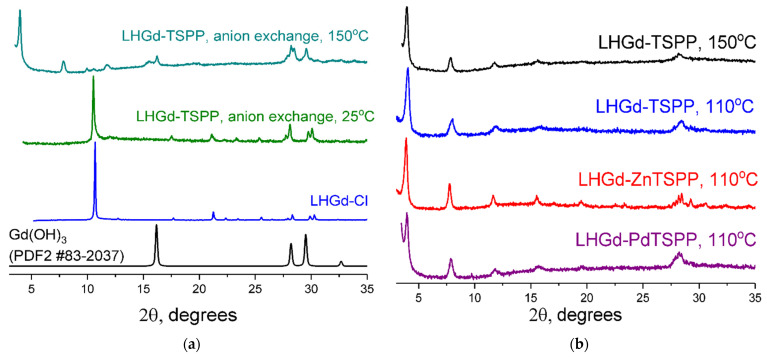
Powder XRD patterns of (**a**) LHGd-Cl and the products of TSPP intercalation into layered gadolinium hydroxide by the anion exchange method at different temperatures; (**b**) LHGd-TSPP, LHGd-ZnTSPP, and LHGd-PdTSPP prepared by the coprecipitation method.

Firstly, layered gadolinium hydroxochloride **LHGd-Cl** was prepared [59]. According to XRD data (Figure 1a), the **LHGd-Cl** obtained crystallised in an orthorhombic system with unit cell parameters a = 12.88, b = 6.39, c = 8.45 Å, which coincided with the diffraction data for gadolinium hydroxochloride Gd_2_(OH)_5_Cl·nH_2_O [24].

The synthetic procedure based on anionic exchange was not successful with *meso*-tetra(4-sulfonatophenyl)porphyrin **TSPP** at 25 °C (Figure 1a). The increase in the anion-exchange reaction temperature to 150 °C allowed for the formation of the layered phase with a basal spacing of 22.6 Å vs. the initial 8.45 Å for **LHGd-Cl** (Figure 1a). The value of the interlayer spacing obtained correlates well with similar values for LDH (23–24 Å) [60] and with the layered hydroxides of europium and terbium (22.4–23.3 Å) [56] intercalated with various bulky porphyrin molecule derivatives. However, according to the XRD pattern, the crystalline phase of gadolinium hydroxide Gd(OH)_3_ was also formed (№ 83-2037 in PDF2 database).

In the case of in situ **TSPP** intercalation (coprecipitation method), a single-phase **LHGd-TSPP** with a basal interplanar distance equal to 22.3 Å was formed (Figure 1b). It should be noted that the decrease in the reaction temperature from 150 to 110 °C caused no changes in the diffraction pattern of the material (Figure 1b). For this reason, the intercalation of **ZnTSPP** and **PdTSPP** metal complexes in layered gadolinium hydroxide was performed at 110 °C. The diffraction patterns of thus obtained phases are quite similar and contain a series of 00l reflexes, confirming the successful intercalation of porphyrins (Figure 1b). Interestingly, a significant (approximately three-fold) difference in particle sizes along the 00l direction was observed for the basic gadolinium chloride synthesized at 95 °C and for the layered gadolinium hydroxides synthesized at 110 °C or 150 °C and intercalated with **TSPP**, **ZnTSPP**, or **PdTSPP**. The estimations were made using the Scherrer equation resulting in the following crystal size values: 77 nm (**LHGd-Cl**); 23 nm (**LHGd-TSPP**, synthesized at 110 °C); 28 nm (**LHGd-TSPP**, synthesised at 150 °C); 27–28 nm (**LHGd-ZnTSPP** and **LHGd-PdTSPP**, both synthesized at 110 °C). These results indicate that, despite the higher temperature of the synthesis, the presence of porhyrin anions in the reaction mixtures hinders the growth of well-crystallised layered gadolinium hydroxide particles. This effect is presumably due to the large size of these anions and their possible diverse coordination modes. Recently, a similar effect was observed for the layered yttrium hydroxides intercalated with *closo*-dodecaborate anion [61].

**Figure 2:**
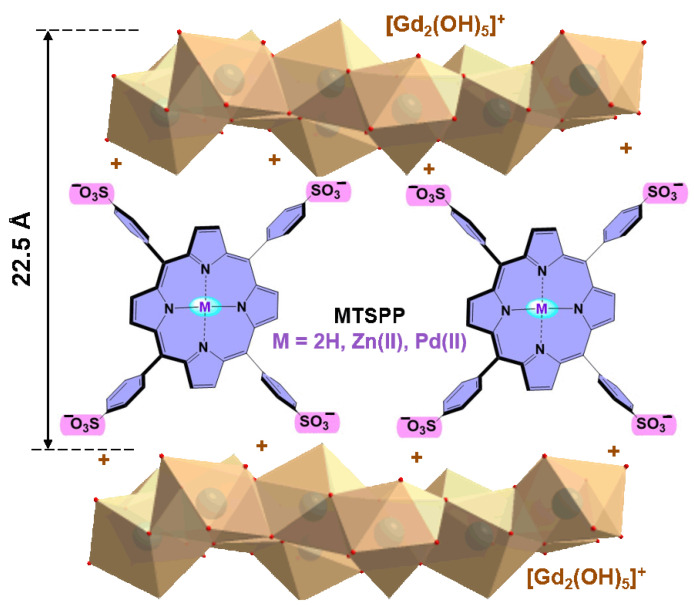
The schematic representation of the anionic 5,10,15,20-tetrakis-(4-sulfonatophenyl)-porphyrins intercalated in the layered gadolinium hydroxide **LHGd**.

The identical positions of the reflexes in this series indicate that the interplanar basal distances were the same for the layered gadolinium hydroxides obtained (~22.5 Å). The schematic representation of the **LHGd-MTSPP** structure is shown in Figure 2.

**Figure 3:**
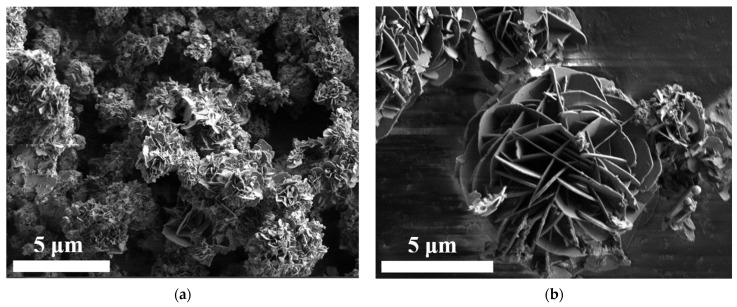
SEM images of (**a**) **LHGd-TSPP** obtained at 150 °C and (**b**) **LHGd-ZnTSPP** obtained at 110 °C.

The microstructure of **LHGd-MTSPP** samples obtained by the coprecipitation method was analysed by SEM. All the materials obtained consisted of individual lamellar particles of 20–50 nm thickness (Figure 3, and Figure S2 in the ESI), which is also characteristic of layered rare-earth hydroxides. The thickness values agree well with the size of coherent scattering domains for this sample (66 nm), calculated by full profile analysis of the diffraction pattern (position of the first reflection, 3.944°2θ, and its width at half-height, 0.1339°2θ).

The comparison of FT-IR spectra of free **TSPP** and intercalated layered hydroxide **LHGd-TSPP** confirmed the presence of the sulfonated porphyrin moieties in this hybrid material (see ESI, Figure S3). In the spectrum of the **LHGd-TSPP** sample, vibrations of –SO_3_^–^ groups, ν_as_(SO_3_^–^) at 1167 cm^–1^ and ν_s_(SO_3_^–^) at 1037 cm^–1^ can be observed, whereas the pure **TSPP** FT-IR-spectrum shows these bands at 1171 cm^–1^ and 1035 cm^–1^, respectively. Similar positions for –SO_3_^−^ stretching bands (1171 cm^–1^ and 1036 cm^–1^) have previously been observed for LDH materials intercalated with sulfonated porphyrins [53]; it was suggested that, in this case, –SO_3_^–^ groups of tetrapyrrole molecules did not directly coordinate metal atoms of the inorganic host [53,54,62,63].

**Figure 4:**
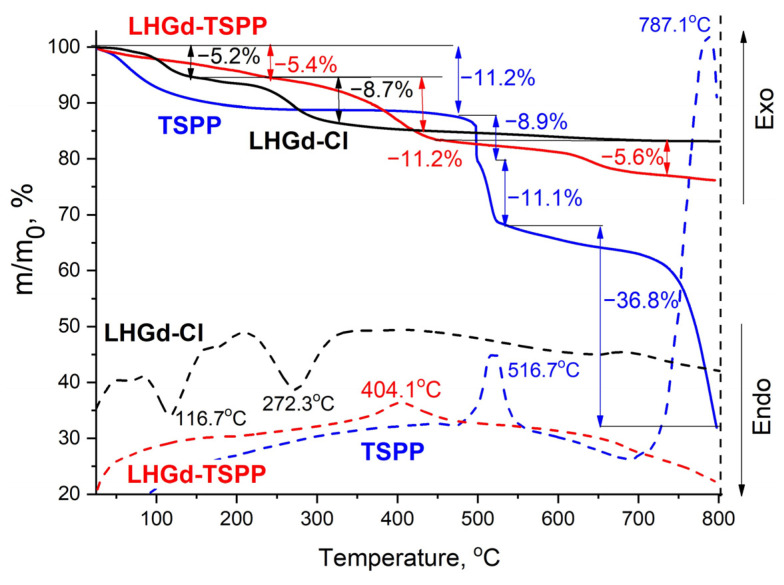
Thermal analysis data for **TSPP** (blue lines)**, LHGd-Cl** (black lines), and **LHGd-TSPP** (red lines).

The intercalation of organic compounds can change the thermal behaviour of the resulting material [64,65,66,67]. To check this, thermogravimetric analysis (TA) was conducted for the **LHGd-TSPP** sample and pure **TSPP** (C_44_H_26_N_4_Na_4_(SO_3_)_4_·12H_2_O, M = 1239 g/mol) and **LHGd-Cl** (Gd_2_(OH)_5_Cl·nH_2_O, M = 462 g/mol). The decomposition of free porphyrin in air proceeded in several stages (Figure 4): at the first stage, at temperatures up to ~200 °C, the loss of physically adsorbed and crystallisation water took place; then, at ~500–530 °C, desulphurisation occurred [68], with the formation of sodium sulfate, accompanied by the oxidation of phenyl moieties of the porphyrin, which was indicated by the exothermic effect at ~520 °C. A further well-defined exothermic effect, with a maximum at 787 °C, was observed on the DTA curve of the tetrasulfoporphyrin sample; it was accompanied by a significant weight loss (more than 36%). According to previously reported data, this effect could be attributed to the removal of phenyl fragments of porphyrin [68].

The thermal behaviour of **LHGd-Cl** (Figure 4) is in line with the previously reported data [69]. The first decomposition stage is attributed to the elimination of interlayer water molecules, and the second stage corresponds to dehydration of Gd_2_(OH)_5_Cl and the formation of Gd_2_O_3_ and GdOCl. The weight loss values associated with these stages (5.2% and 8.9%, respectively) are in a good agreement with the calculated ones (5.8% and 9.7%, respectively).

The expected composition of **LHGd-TSPP** hybrid material was [Gd_2_(OH)_5_]_4_TSPP·nH_2_O, assuming that chloride ions were completely substituted with porphyrin. Firstly, **LHGd-TSPP** showed a weight loss of 5.4% up to 230 °C, which was probably due to the loss of crystallisation water. Taking the molar mass of the anhydrous sample [Gd_2_(OH)_5_]_4_TSPP to be equal to 2526 g/mol and considering the loss of **x** crystallisation water molecules in [Gd_2_(OH)_5_]_4_TSPP·xH_2_O to be equal to 5.4%, **x** can be calculated as ≈ 8. Thus, the composition of **LHGd-TSPP** corresponds to the formula [Gd_2_(OH)_5_]_4_TSPP·8H_2_O, M = 2670 g/mol. Weight loss at the second stage (11.2% of the sample weight) in the temperature range 230–440 °C may have been due to the dehydration of the layered hydroxide itself. Interestingly, the first and the second stages of **LHGd-TSPP** decomposition proceed at higher temperatures (by 50–100 °C) than these of **LHGd-Cl**. This effect presumably evidences the higher thermal stability of the layered hydroxide host containing porphyrin moieties modified with sulfo-groups. Generally, layered rare-earth hydroxides intercalated with sulfates possess a higher thermal stability than those intercalated with nitrates or chlorides [70]. Note that the total weight loss for **LHGd-TSPP** at stages II and III was 16.8%, i.e., about 449 g/mol, which was much less than the molar weight of the intercalated porphyrin (930 g/mol); therefore, the intercalated porphyrin did not fully decompose, even after heating to 800 °C. However, the exothermic effect at 404 °C may indicate the partial decomposition of intercalated porphyrin with the release of CO_2_, NO_2_, SO_2_, and H_2_O, as has been shown previously for layered europium and terbium hydroxides with intercalated sulfoporphyrins [56]. It should be noted that, according to previously reported data [56], layered europium hydroxide intercalated with tetrasulfoporphyrin loses about 40% of its weight at 600 °C, while **LHGd-TSPP** showed only a 22.2% weight loss at 800 °C, which confirms the higher thermal stability of the **LHGd-TSPP** material.

CHNS analysis data were in good agreement with the elemental composition calculated using the formula [Gd_2_(OH)_5_]_4_[C_44_H_26_N_4_(SO_3_)_4_]_3/4_Cl·8H_2_O, in which 75% of the chloride anions were substituted by porphyrin molecules (ESI, Table S1).

EDX data for **LHGd-TSPP** (Table 1) agree well with the CHNS analysis results, confirming the [Gd_2_(OH)_5_]_4_[C_44_H_26_N_4_(SO_3_)_4_]_3/4_Cl·8H_2_O composition. According to EDX results for **LHGd-PdTSPP**, the measured **PdTSPP** content was less than the calculated value, which means that chloride ions were not fully replaced by palladium(II) porphyrinates. In the case of **LHGd-ZnTSPP**, the smallest element ratio deviations were observed, so the composition of this material was very close to [Gd_2_(OH)_5_]_4_ZnTSPP·nH_2_O.

### 2.2. Optical Properties of the LHGd Intercalated with Porphyrin

The ground state absorption spectra of solid porphyrin-**LHGd** were measured using UV-Vis diffuse reflectance spectroscopy. The resulting spectra of the composites exhibit Soret and Q absorption bands characteristic of porphyrins, confirming the identity of the guest molecules (ESI, Figure S4). There is some broadening of the Soret band for intercalated **MTSPP** (M **=** 2H, Zn(II) and Pd(II)) species, compared with the sharp Soret band of the corresponding porphyrins in aqueous solutions. These spectral features can be attributed to the interactions between the periphery of individual molecules and the densely packed hydroxide layers through a range of different orientations of the porphyrin macrocycles. Similar results have previously been reported for sulfoporphyrin intercalated LDH materials [50,54,55].

**Figure 5:**
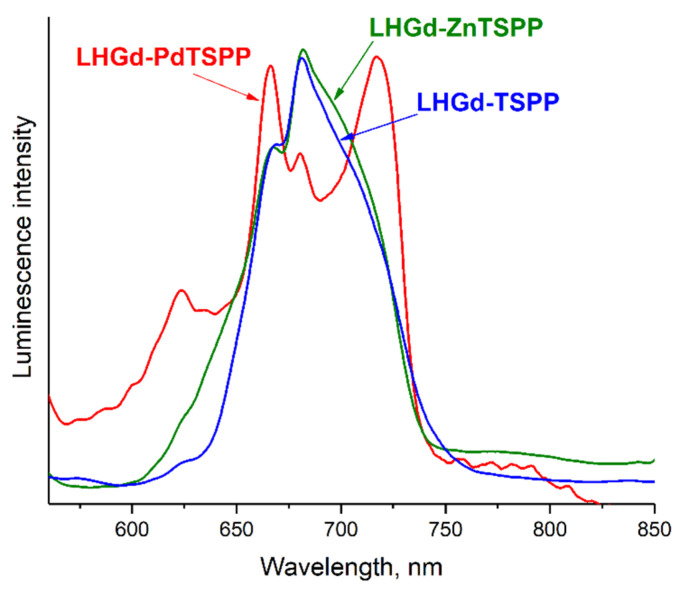
Normalised luminescence spectra of **LHGd-MTSPP** samples excited at 520 nm.

All **LHGd-MTSPP** (M **=** 2H, Zn(II) and Pd(II)) hybrids exhibit luminescence caused by intercalated porphyrins, when excited at 520 nm (Figure 5). In the emission spectrum of **LHGd-TSPP**, there is a major luminescence peak at 681 nm, with a shoulder at 667 nm. These bands are red-shifted relative to the Q (0,0) (645 nm) band and blue-shifted relative to the Q (0,1) (703 nm) band of the individual **TSPP** porphyrin in aqueous solution. There is also an increase in the Q (0,1) band intensity, which may indicate the interaction between host and guest molecules. Interestingly, two fluorescence bands of **LHGd-ZnTSPP** are almost the same as those of **LHGd-TSPP** (667 nm and 681 nm), although the emission spectrum of the **ZnTSPP** aqueous solution shifts to a shorter wavelength region (606 nm and 655 nm, respectively) [71,72]. After intercalation of the **PdTSPP** complex into **LHGd**, it also remains photoactive, with a fluorescence emission band at 623 nm and phosphorescence emission bands at 666 nm and 717 nm (Figure 5). The intensity ratio of phosphorescence/fluorescence in the hybrid material changed in comparison with free **PdTPPS**, due to the suppressed quenching of the porphyrin triplet states by oxygen in the interlayer space of **LHGd**. Similar effects have previously been reported for **PdTPPS** intercalated in **MgAl LDH** [55]. Thus, the photophysical properties of **LHGd-MTSPP** hybrids (M = 2H, Zn(II) and Pd(II)) confirm the intercalation of porphyrin molecules in the **LHGd** interlayer space; they do not interact with one another in either ground or triplet states, which makes the composites photoactive.

### 2.3. Radical Scavenging Properties of the LHGd Intercalated with TSPP, ZnTSPP, and PdTSPP

The radical scavenging properties of hybrid materials consisting of layered gadolinium hydroxide and intercalated sulfoporphyrins were studied by monitoring their impact on luminol (3-aminophtalhydrazide) chemiluminescence in the presence of the alkylperoxyl radical source ABAP (2,2′-azobis(2-amidinopropane)dihydrochloride) in phosphate buffer solution (pH 7.4). The radical scavenging properties of individual components of hybrid materials, namely **LHGd-Cl** sol and solutions of free **TSPP**, **ZnTSPP**, and **PdTSPP**, were also investigated to reveal the mutual influence of the layered host matrix and the guest dye molecules. The dynamic light scattering data for the layered gadolinium hydroxide are given in ESI, Figure S5.

**Figure 6:**
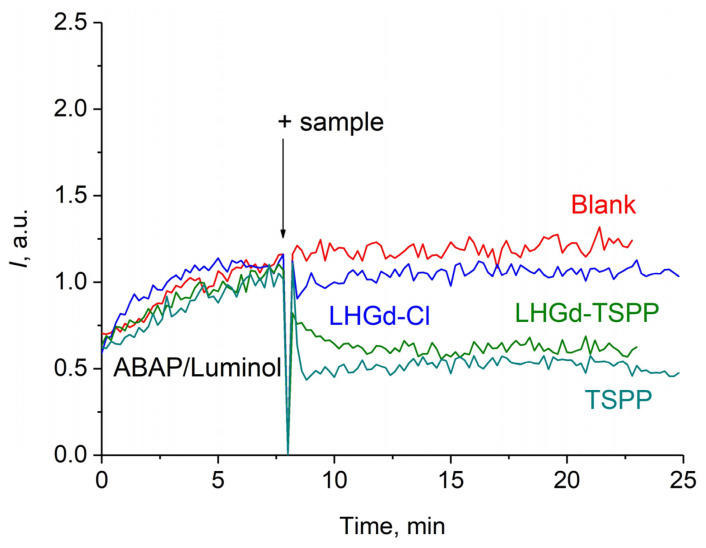
Chemiluminescence curves for **LHGd-Cl** (10 μM), **LHGd-TSPP** (10 μM), and **TSPP** (4 μM) sols in the ABAP/luminol system in phosphate buffer solution (pH 7.4).

Chemiluminescence curves for **LHGd-TSPP** sol, as well as for individual **LHGd-Cl** and **TSPP** solutions, are shown in Figure 6. The addition of a **LHGd-TSPP** hybrid and the corresponding free **TSPP** to the ABAP/luminol system in phosphate buffer solution led to a significant decrease in luminescence intensity.

The observed kinetics is characteristic of prolonged-action antioxidants, i.e., substances scavenging free radicals at a relatively low rate. To compare the antioxidant activities of the substances, the following quantitative measure was chosen: concentration of chemiluminescence semi-suppression (c_0.5_, the concentration that reduces twofold the amplitude (*I*) or light sum (*S*) of the chemiluminescent response). The most pronounced inhibitory effect on luminescence was exerted by a solution of free porphyrin and **LHGd-TSPP** sol: c_0.5_ (**LHGd-Cl**) 35 μM, c_0.5_ (**LHGd-TSPP**) 12 μM, c_0.5_ (**TSPP**) 3.5 μM. Since a sol of an individual **LHGd-Cl** did not demonstrate any antioxidant activity, the radical scavenging properties of **LHGd-TSPP** are caused by the presence of intercalated **TSPP** molecules.

**Figure 7:**
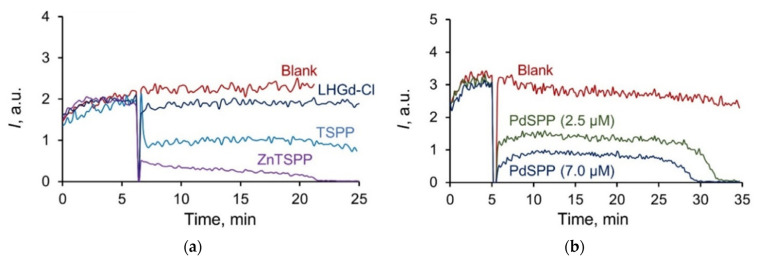
Chemiluminescence curves for (**a**) **LHGd-Cl** sol (10 μM), **TSPP** (4 μM), **ZnTSPP** (0.4 μM), and (**b**) **PdTSPP** in the ABAP/luminol system in phosphate buffer solution (pH 7.4).

Analysis of luminol chemiluminescence in the presence of ROS generated by ABAP revealed that Zn(II) and Pd(II) complexes with 5,10,15,20-tetra-(4-sulfonatophenyl)porphyrin exhibit similar, but more pronounced, antioxidant properties compared with the corresponding porphyrin (Figure 7). The luminescence semi-suppression concentrations were calculated for the samples analysed, as follows: c_0.5_ (**TSPP**) 3.5 μM, c_0.5_ (**ZnTSPP**) 0.1 μM, and c_0.5_ (**PdTSPP**) 2.2 μM. According to these data, **ZnTSPP** has the highest radical-scavenging activity, which correlates well with the value of the anodic potentials for the investigated porphyrins [73]. The observed effect was dose-dependent, as demonstrated for the palladium complex (Figure 7b), which makes it possible to tune the antioxidant activity by varying the concentration of the substances.

**Figure 8:**
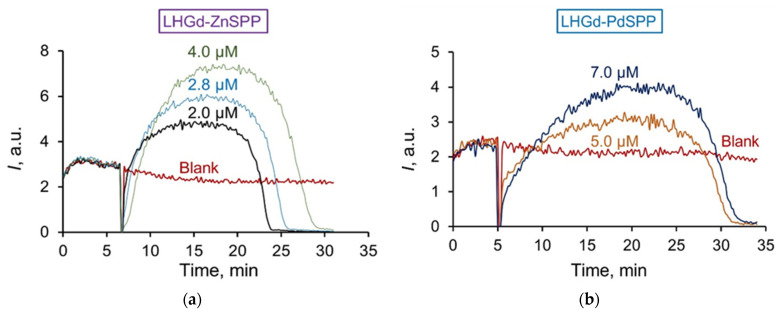
Chemiluminescence curves of (**a**) **LHGd-ZnTSPP** and (**b**) **LHGd-PdTSPP** sols in the ABAP/luminol system in phosphate buffer solution (pH 7.4).

Chemiluminescence kinetics for **LHGd-ZnTSPP** and **LHGd-PdTSPP** sols (Figure 8) differed significantly from that of **LHGd-TSPP** and **MTSPP** samples. The addition of **LHGd-ZnTSPP** or **LHGd-PdTSPP** to the ABAP/luminol system at pH 7.4 led to an increase in the luminescence intensity relative to the control level. Thus, the intercalation of **ZnTSPP** and **PdTSPP** species into the **LHGd** matrix resulted in their activity changing from being antioxidant, to being prooxidant. The observed prooxidant action was also dose-dependent. It has recently been shown that, in some cases, metalloporphyrins exhibit both antioxidant [42,74,75,76] and prooxidant properties [46,47,77,78], depending on environmental conditions, such as the presence of the other reagents in the system [44], the level of reactive oxygen species, the availability of oxygen, etc. [47]. According to the UV-Vis and luminescence spectroscopy data (see Section 3.2), upon intercalation, the electronic properties of porphyrins changed due to their interaction with the densely packed metal hydroxide layers. Suppressed quenching of **PdTSPP** triplet states by oxygen upon intercalation in **LHGd** was demonstrated in Section 3.2. This indicates the low availability of oxygen in the prepared hybrid materials in comparison with aqueous solutions of metal porphyrins. Moreover, as a result of the intercalation process, the porphyrins were fixed in a matrix containing a large number of hydroxo-groups. To check the possible influence of pH on the radical-scavenging properties, **ZnTSPP** behaviour was analysed in a highly alkaline (pH 12) solution (see ESI, Figure S6). The data obtained show a significant prooxidant effect of the zinc(II) complex. Thus, it is suggested that a combination of factors, such as changes in local environment/electronic properties and the low availability of oxygen in the interlayer space, could have caused the switch of radical-scavenging activity of intercalated **ZnTSPP** and **PdTSPP** metalloporphyrins.

Thus, in this study, it was established that **ZnTSPP, PdTSPP**, and **LHGd-TSPP** act as an antioxidant towards alkylperoxyl radicals. At the same time, the intercalation of **ZnTSPP** and **PdTSPP** in the gadolinium hydroxide layered matrix inverts their biochemical activity from being antioxidant to being prooxidant. The data obtained indicate that hybrid materials based on porphyrins and LRHs can be considered as nanozymes with tunable activity. These results suggest possible biomedical applications of hybrid materials based on biocompatible porphyrins intercalated in a low-soluble inert matrix, e.g., in cytotoxic therapy or in the regulation of redox homeostasis disorders.

## 3. Materials and Methods

### 3.1. Materials

The following reagents were used for the syntheses, without additional purification: NaCl (Chemmed, chemical pure), hexamethylenetetramine (99%, Alfa Aesar, Ward Hill, MA, USA), GdCl_3_·6H_2_O (99.9%, Lanhit, Moscow, Russia), tetrasodium 5,10,15,20-*meso*-tetra-(4-sulfonatophenyl)porphyrin dodecahydrate (95%, Alfa Aesar, Ward Hill, MA, USA), zinc(II) acetate dihydrate (≥95%, LenReactiv, Saint-Petersburg, Russia), palladium(II) acetate (98%, Acros Organics, Pittsburgh, PA), and N,N-dimethylformamide (99.8%, Sigma-Aldrich, Saint-Louis, MO, USA).

### 3.2. Porphyrin Synthesis

Zinc(II) and palladium(II) 5,10,15,20-*meso*-tetra-(4-sulfonatophenyl)porphyrinates **ZnTSPP** and **PdTSPP**, respectively, were obtained according to a previously published protocol [73,79], which included metalation of the free base porphyrin **TSPP** with the corresponding metal(II) acetate in boiling N,N-dimethylformamide. The resulting **ZnTSPP** and **PdTSPP** complexes were purified by gel filtration using Sephadex LH-20 resin.

**ZnTSPP**: ^1^H NMR (600 MHz, DMSO, 25 °C): *δ*_H_ 8.78 (8H, s, H*β*), 8.13 (8H, d, ^3^*J*_H,H_ = 8.1 Hz, *o*-Ph), 8.02 (8H, d, ^3^*J*_H,H_ = 8.1 Hz, *m*-Ph). UV-Vis (H_2_O; *λ*_max_, nm): 420, 557, 597.

**PdTSPP**: ^1^H NMR (600 MHz, DMSO, 25 °C): *δ*_H_ 8.81 (8H, s, H*β*), 8.14 (8H, d, *^3^J*_H,H_ = 8.1 Hz, *o*-Ph), 8.03 (8H, d, *^3^J*_H,H_ = 8.1 Hz, *m*-Ph). UV-Vis (H_2_O; *λ*_max_, nm): 412, 522.

### 3.3. Gd(III) Layered Hydroxochloride (LHGd-Cl) Synthesis

Layered gadolinium hydroxochloride **LHGd-Cl** was prepared according to a previously reported procedure [59,80] and used as a host for the intercalation of porphyrin molecules by the anion exchange procedure described below. For **LHGd-Cl** synthesis, the following solutions in deionised water were prepared: 0.113 M GdCl_3_·6H_2_O, 0.171 M hexamethylenetetramine (HMT), and 1.2 M NaCl. 120 mL of GdCl_3_·6H_2_O solution was mixed with 100 mL of HMT solution and 100 mL of NaCl solution under continuous stirring. HMT is a commonly used reagent for homogeneous hydrolysis [81]; NaCl was added to maintain ionic strength during chloride-anions intercalation. Ultrasonic treatment was used to prevent the formation of basic rare-earth carbonates. The total solution volume was brought to 1.5 L with deionised water. To initiate HMT hydrolysis, the mixture was heated at 95 °C for 1 h under continuous stirring. The resulting **LHGd-Cl** precipitate was separated on a glass filter, washed three times with deionised water, and then dried at 50 °C and with a relative humidity of 75%. **LHGd-Cl** (1.819 g, a total yield of 58 wt.%) was obtained as a white crystalline powder. According to EDX data, the chemical formula of the substance was Gd_2_(OH)_5.09_Cl_0.91_.

### 3.4. Preparation of LHGd-Porphyrin Hybrid Materials (LHGd-MTSPP)

#### 3.4.1. General Procedure for Anion Exchange Reactions

For the synthesis of porphyrin-intercalated **LHGd-MTSPP** (M = 2H, Zn(II) and Pd(II)) samples, a reaction was carried out between **LHGd-Cl** and the anionic *meso*-tetra(4-sulfonatophenyl)porphyrin **MTSPP** (M **=** 2H, Zn(II) and Pd(II)). The **LHGd-Cl** powder was dispersed in an aqueous solution of porphyrin (0.2 mM) and the Gd_2_(OH)_5_Cl:porphyrin molar ratio was adjusted to 4:1 (assuming that four Cl^-^ anions are replaced by one four-charged porphyrin anion). A pH of 6.8–7.0 was maintained by the addition of 0.1 M KOH_(aq)_. The mixture was stirred at room temperature for 10 min. The suspension was hydrothermally (HT-) treated at 150 °C in a Teflon autoclave for 24 h. Then, a coloured solid product was filtered off, washed with distilled water, and dried at 50 °C for 24 h.

#### 3.4.2. General Procedure for the Coprecipitation Method

Aqueous solutions of **MTSPP** (M **=** 2H, Zn(II) and Pd(II)) porphyrin (0.8 mM, 35 mL) and GdCl_3_ (0.113 M, 2 mL, 8-fold molar excess) were mixed under stirring. Then, an aqueous solution of HMT (0.057 M, 5 mL) was added and the total solution volume was adjusted to 50 mL with distilled water. The reaction mixture was placed in a 100 mL Teflon autoclave and subjected to hydrothermal treatment at 110–150 °C for 24 h. The coloured precipitate obtained was filtered, washed with distilled water, and dried at 50 °C for 24 h.

The products of intercalation of **TSPP**, **PdTSPP**, and **ZnTSPP** into layered gadolinium hydroxide were designated as **LHGd-TSPP**, **LHGd-ZnTSPP**, and **LHGd-PdTSPP**, respectively. The intercalation process was monitored by UV-Visible spectroscopy by detecting a decrease in the absorption intensity of porphyrin solution in the reaction mixture (Figure S1, ESI).

### 3.5. Instrumental Methods

Powder X-ray diffraction analysis (XRD) was performed on a Bruker (Billerica, MA, USA) D8 Advance diffractometer (CuK_α_-radiation, Ni filter) operating in Bragg–Brentano geometry and equipped with a LYNXeye detector. All diffraction patterns were registered in the range of 2.5–50°2θ at 0.02°2θ step and with a signal accumulation time of 0.05 sec per point. Crystallite sizes were estimated using the Scherrer equation for 00l diffraction maxima [82]. A full-profile description of the diffraction patterns was performed using the Fityk software v.1.3.1 [83].

Energy dispersive X-ray analysis (EDX) and scanning electron microscopy (SEM) of the samples were performed using a Carl Zeiss NVision 40 microscope (Oberkochen, Germany) equipped with an Oxford Instruments X-Max analyser (GB) at accelerating voltages of 20 and 1 kV, respectively.

FT-IR spectra of the powders were recorded on a Bruker ALPHA device in the mode of attenuated total reflectance.

Thermogravimetric analysis (TGA) of the samples was carried out in air and argon using a TA Instruments SDTQ600 thermo analyser. The sample weights were 10–20 mg. The heating to 800 °C was performed at 10 deg/min in a synthetic air flow (250 mL/min).

CHNS-elemental analysis was performed on a EuroVector EA3000 (Pavia, Italy) elemental analyser in a temperature range up to 900 °C.

Diffuse reflectance spectra of solid samples were recorded on an Evolution 201 spectrophotometer (Thermo Scientific, Waltham, MA, USA) equipped with an integrating sphere. The spectra were converted from reflectance to absorbance using the Kubelka–Munk formula.

Luminescence spectra were recorded using an Ocean Optics system (Orlando, FL, USA) with a monochromator; the measurements were performed in the range of 500–800 nm at an excitation wavelength of 520 nm. The wavelength was chosen to avoid instrument off-scale readings due to the extremely high intensity of porphyrins’ luminescence.

Particle size distribution in aqueous sols was analysed by dynamic light scattering (DLS), using a Photocor Complex multi-angle spectrometer (Photocore Ltd., Moscow, Russia) with a diode laser (λ = 650 nm, emission power 25 mW).

The chemiluminescent analysis was performed using the aqueous sols of the materials obtained (**LHGd-TSPP**, **LHGd-ZnTSPP**, **LHGd-PdTSPP**, and free **LHGd-Cl** as a comparison sample). For **LHGd-MTSPP** sol preparation, 1 mg of the corresponding sample was suspended in 10 mL of distilled water, followed by sonication for 20 min. The results of DLS (see Figure S5) showed that the hydrodynamic particle diameters in **LHGd-TSPP**, **LHGd-ZnTSPP**, and **LHGd-PdTSPP** sols were 290 ± 80 nm, 220 ± 79 nm, and 282 ± 72 nm, respectively. According to DLS data, the hydrodynamic diameter of the particles remained unchanged for at least six days, indicating the good stability of the sols obtained.

Chemiluminescence was recorded using a 12-channel Lum-1200 chemiluminometer (DISoft, Moscow, Russia). A steady-state chemiluminescence signal was generated as a result of alkylperoxyl radical formation during thermolysis of 2,2′-azobis(2-amidinopropane)dihydrochloride (ABAP, Sigma #440914; 2.5 μM) in the presence of a chemiluminescence probe, luminol (5-amino-1,2,3,4-tetrahydro-1,4-fthalazindione, 3-aminophthalic acid hydrazide, Sigma #123072; 2.0 μM), in potassium-phosphate buffer solution KH_2_PO_4_ (Sigma #1.04873), 100 mM, pH 7.4) at 37 °C. The total volume of the system was 1.000 mL.

## 4. Conclusions

In this work, a series of new hybrid materials with enzyme-like properties were successfully prepared by intercalation of tetrasulfophenyl porphyrin **TSPP** and its Zn(II) and Pd(II) complexes (**ZnTSPP**, **PdTSPP**) into layered gadolinium hydroxide by co-precipitation during hydrothermal treatment. The layered gadolinium hydroxide (LRH) intercalated with **TSPP** species showed a higher thermal stability, as demonstrated by TA data. For the first time, the radical scavenging properties of the sulfoporphyrins **MTSPP** (M **=** 2H, Zn(II), Pd(II)) and hybrid materials prepared by the intercalation of these species into LRHs were investigated using the chemiluminescence method. In contrast to the free porphyrin molecules, which possessed antioxidant properties, biochemical activity towards the alkylperoxyl radical of **LHGd-MTSPP** (M **=** 2H, Zn(II), Pd(II)) was found to depend on the type of the intercalated porphyrin. **LHGd-TSPP** acted as a typical antioxidant of prolonged action. At the same time, intercalation of **ZnTSPP** and **PdTSPP** in the gadolinium hydroxide matrix led to their biochemical behaviour changing to being a dose-dependent prooxidant activity. It was suggested that a combination of factors, such as changes in the local environment/electronic properties and the low availability of oxygen in the interlayer space, could influence the inversion of the radical-scavenging activity of intercalated metal porphyrins. Therefore, this paper provides an effective approach to the design and development of new hybrid LRH-porphyrin materials that show promise as nanozymes with switchable activity.

## Figures and Tables

**Table 1:** Element ratios in **LHGd-TSPP**, **LHGd-ZnTSPP**, and **LHGd-PdTSPP**, according to EDX data.

Material	Element Ratio	Calculated	Found
[Gd_2_(OH)_5_]_4_TSPP_3/4_Cl nH_2_O	Gd:S	2.7:1	2.6:1
[Gd_2_(OH)_5_]_4_PdTSPP·nH_2_O	Gd:Cl	∞	6.6:1
	Gd:S	2:1	2.3:1
	Gd:Pd	8:1	10.0:1
	S:Pd	4:1	4.3:1
[Gd_2_(OH)_5_]_4_ZnTSPP·nH_2_O	Gd:Cl	∞	56.1:1
	Gd:S	2:1	1.7:1
	Gd:Zn	8:1	7.6:1
	S:Zn	4:1	4.5:1

## Data Availability

The data generated in the present study are available from the corresponding author upon reasonable request.

## Supplementary Materials

The following supporting information can be downloaded at: https://www.mdpi.com/article/10.3390/ijms232315373/s1.
