# Supplementary material for: Switchable Nanozyme Activity of Porphyrins Intercalated in Layered Gadolinium Hydroxide
Source: Int J Mol Sci. 2022 Dec 6;23(23):15373. doi: 10.3390/ijms232315373 (PMC9736057; doi:10.3390/ijms232315373)
Supplement: Supplementary file 1 [file ijms-23-15373-s001.zip › ijms-2015066-supplementary.pdf]

Electronic Supporting Information

Switchable Nanozyme Activity of Porphyrins Intercalated in Layered Gadolinium Hydroxide

Maria A. Teplonogova [1], Marina V. Volostnykh [2], Alexey D. Yapryntsev [1], Madina M. Sozarukova [1], Yulia G. Gorbunova [1], Ekaterina D. Sheichenko [3], Alexander E. Baranchikov [1] and Vladimir K. Ivanov [1,*]

[1] Kurnakov Institute of General and Inorganic Chemistry of the Russian Academy of Sciences, 119991 Moscow, Russia
[2] Frumkin Institute of Physical Chemistry and Electrochemistry of the Russian Academy of Sciences, 119071 Moscow, Russia
[3] Faculty of Chemistry, National Research University "Higher School of Economics", 109028 Moscow, Russia
* Correspondence: van@igic.ras.ru

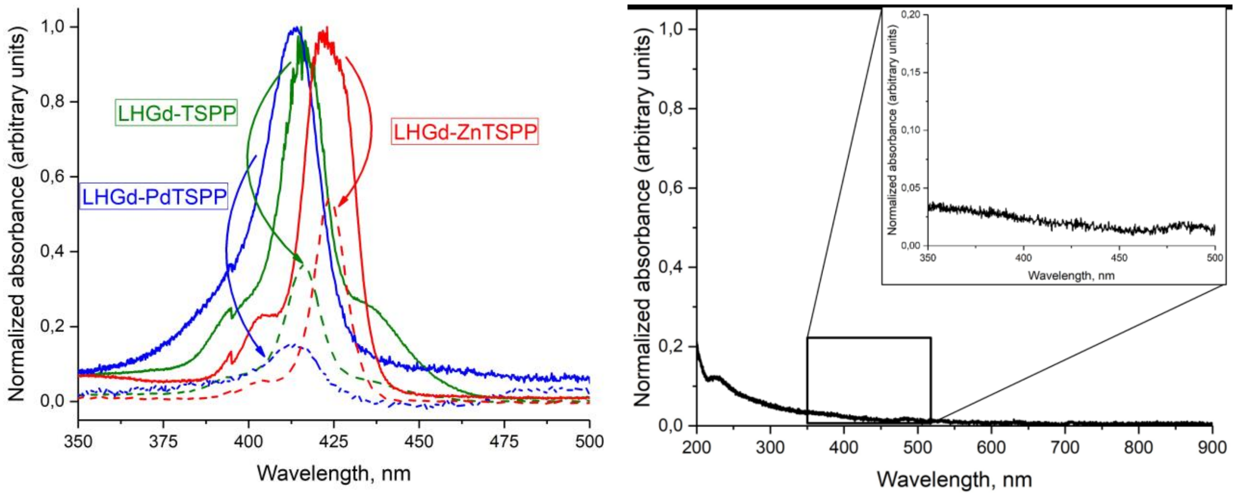

(a) (b)

Figure S1. UV-Visible absorption spectra of (a) aqueous solutions of **TSPP**, **ZnTSPP** and **PdTSPP** before (solid lines) and after (dashed lines) coprecipitation syntheses; (b) **LHGd-Cl** sol (five-fold higher concentration than that of **LHGd-MTSPP**).

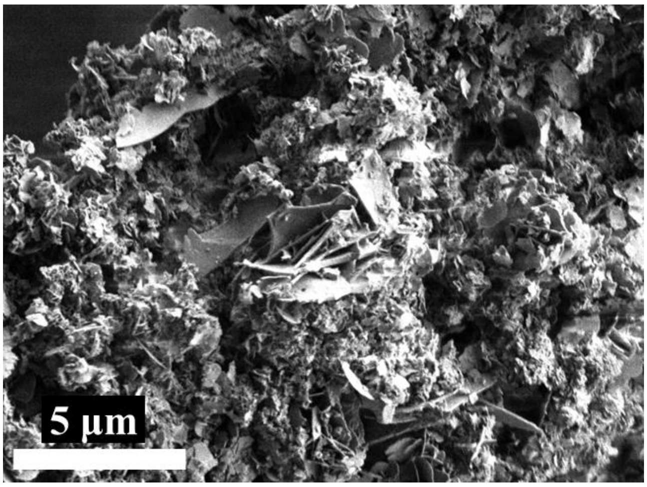

Figure S2. SEM image of **LHGd-PdTSPP** sample obtained by coprecipitation at 110°C.

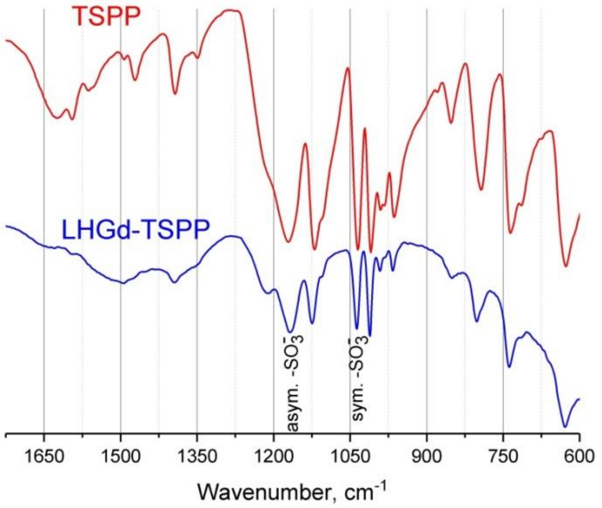

Figure S3. FT-IR spectra of **TSPP** and **LHGd-TSPP**.

Table S1. Comparison of CHNS elemental analysis data and calculated element content in the **LHGd-TSPP** sample.

Formula	Element content, wt. %			
	N	C	H	S
found	1.60	15.86	2.48	3.35
calculated: $[Gd_2(OH)_5]_4C_{44}H_{26}N_4(SO_3)_4 \cdot 8H_2O$	2.10	19.77	2.32	4.79
calculated: $[Gd_2(OH)_5]_4[C_{44}H_{26}N_4(SO_3)_4]_{3/4}Cl \cdot 8H_2O$	1.70	16.01	2.24	3.88

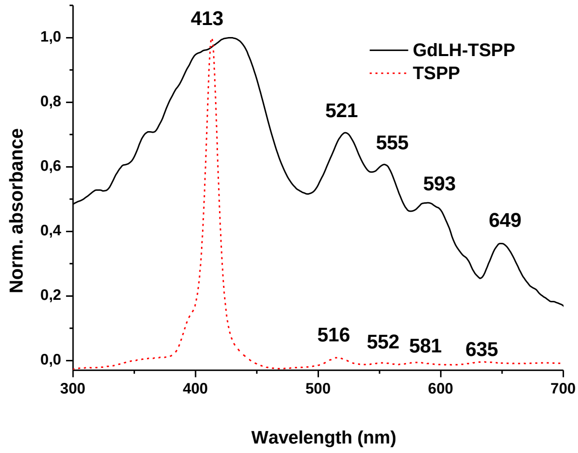

(a)

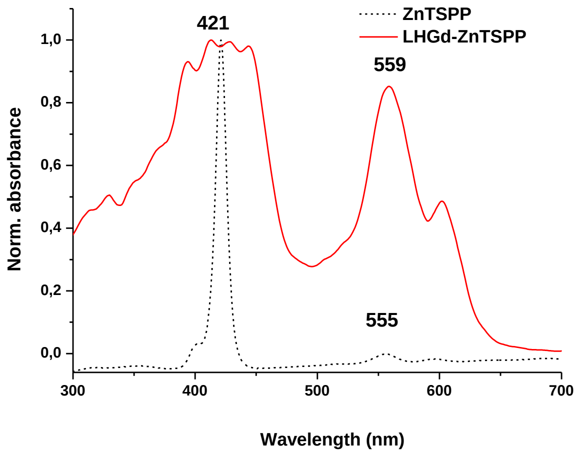

(b)

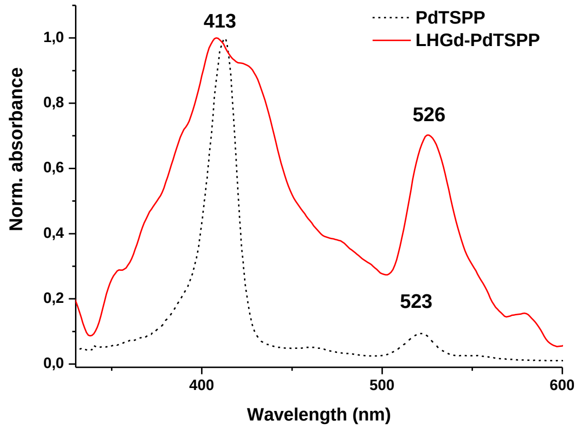

(c)

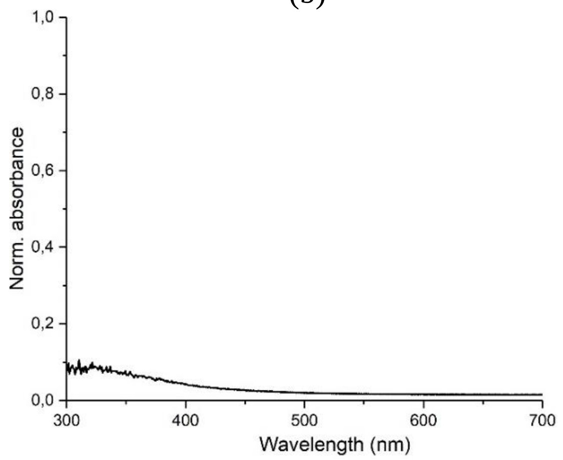

(d)

Figure S4. Normalised diffuse reflectance spectra of **LHGd-TSPP** (a), **LHGd-ZnTSPP** (b), **LHGd-PdTSPP** (c) and **LHGd-Cl** (d) solid samples compared to the absorption spectra of the corresponding porphyrins (dashed line) in aqueous solutions.

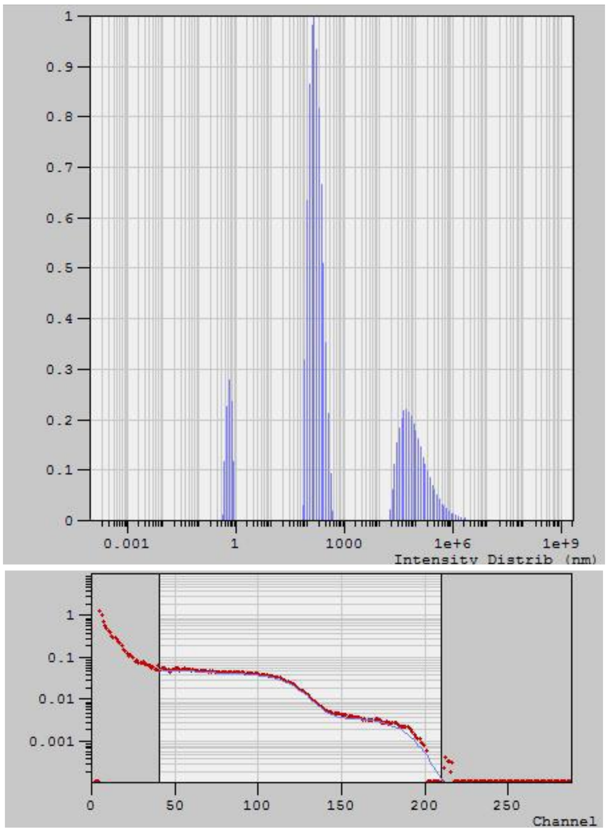

(a)

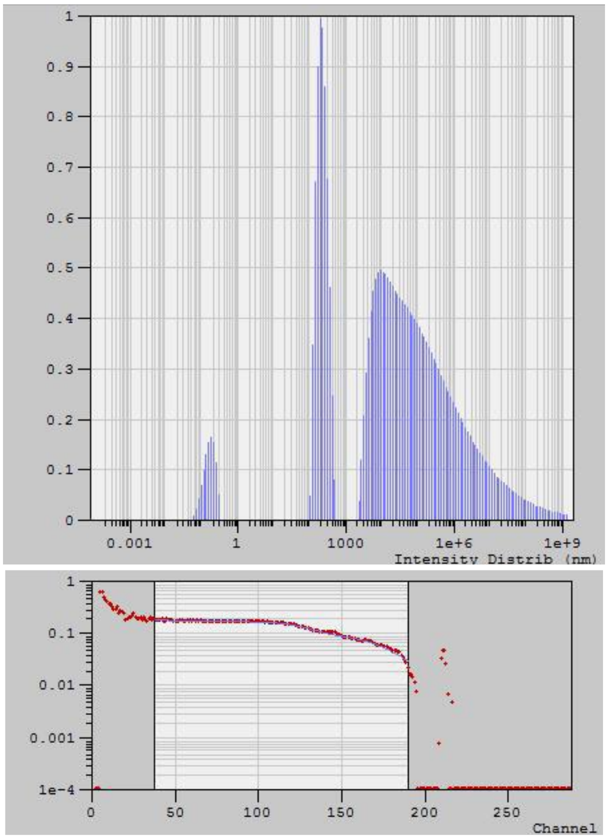

(b)

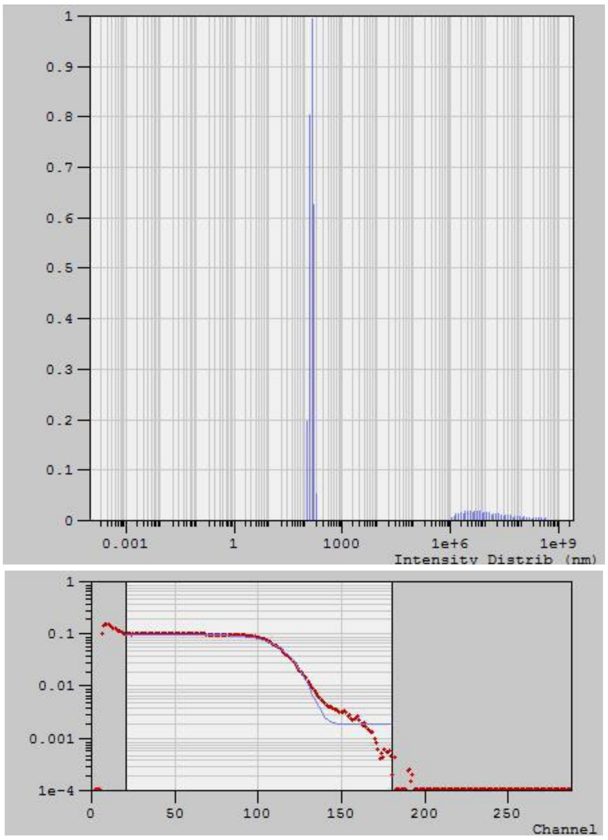

(c)

Figure S5. Dynamic light scattering results for the sols of **LHGd-TSPP** (a), **LHGd-ZnTSPP** (b), and **LHGd-PdTSPP** (c): particle size distributions and correlation functions.

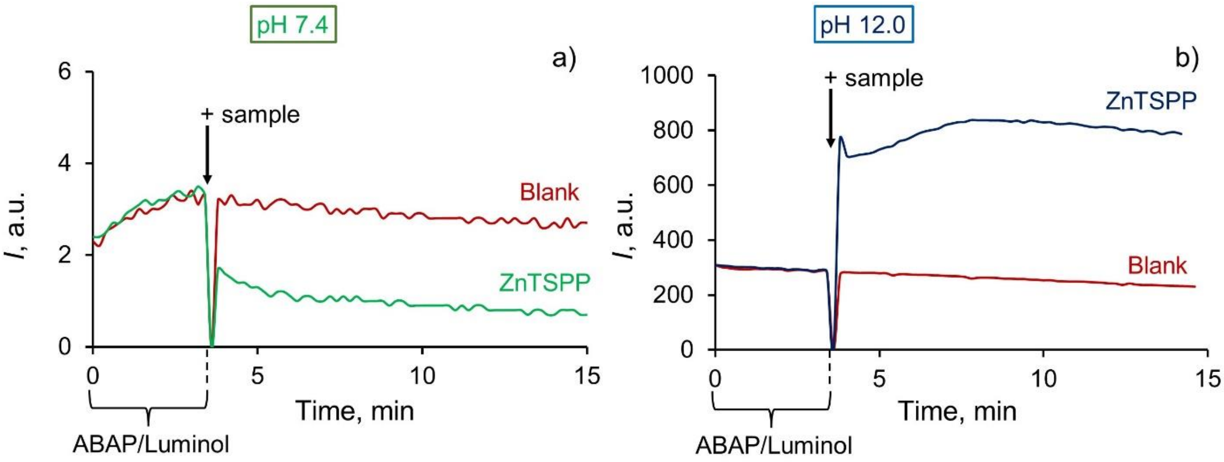

Figure S6. Chemiluminescence curves of ZnTSPP (4 μM) in the ABAP / luminol system in a phosphate buffer solution at (a) pH 7.4, (b) pH 12.0.
